# What can we learn from selfish loci that break Mendel’s law?

**DOI:** 10.1371/journal.pbio.3001700

**Published:** 2022-07-19

**Authors:** Sarah E. Zanders

**Affiliations:** 1 Stowers Institute for Medical Research, Kansas City, Missouri, United States of America; 2 Department of Molecular and Integrative Physiology, University of Kansas Medical Center, Kansas City, Kansas, United States of America

## Abstract

Exceptions to Mendel’s law of segregation were important for demonstrating that chromosomes carry genetic material. Scrutiny of other exceptional inheritance patterns has the potential to unravel unsolved mysteries of genetics.

## Teaser

Exceptions to Mendel’s law of segregation were important for demonstrating that chromosomes carry genetic material. Scrutiny of additional exceptions to Mendel’s law caused by selfish genes has the potential to unravel other unsolved mysteries of genetics.

The heredity of most genes in eukaryotes can be described as “mendelian.” This term is, of course, named after Gregor Mendel, the first to formally describe the foundations of eukaryotic genetics [1]. Mendelian genes are those that follow what is now known as Mendel’s law of segregation. This law describes how parents transmit only 1 of the 2 copies they carry of a given gene to a given offspring. The gene copy selected is random and diploid organisms inherit 1 copy of each gene from each parent.

Mendel deciphered this crucial pattern of heredity long before chromosomes were demonstrated to carry the genetic information. Mendel was, therefore, unaware that his law of segregation reflected the transmission of chromosomes through meiosis. Specifically, diploid organisms (like ourselves) carry 2 sets of chromosomes, 1 set inherited from each parent. To reproduce sexually, eukaryotes use meiosis to generate gametes, like sperm, that contain 1 complete set of randomly selected chromosomes. Each chromosomal locus found in a diploid organism is generally transmitted to half of its gametes. When 2 compatible gametes fuse, a new diploid organism can be created.

Mendel’s law of segregation provides a critical foundation for genetic inquiry. It is not, however, without exceptions. Historically, 2 such exceptions (sex chromosome linkage and chromosome missegregation in meiosis) were used by *Drosophila* geneticists to help demonstrate that genes are carried on chromosomes [2]. Looking forward, modern geneticists interested in understanding the mechanisms of heredity have much to learn from additional exceptions to Mendel’s law.

In particular, selfish genes that break Mendel’s law of segregation to gain a transmission advantage into the next generation are likely to be oversized contributors to shaping the process of sexual reproduction [3]. These selfish genes exploit reproduction such that a given selfish locus is transmitted to more than half of the offspring produced by an organism. There are a variety of selfish DNAs, but here I will focus on transposable elements and drive loci as examples. Transposable elements can generate novel copies of themselves using copy and paste or cut and paste mechanisms. Importantly, transposable elements are selected to mobilize in the germline as that allows new copies to be passed on to subsequent generations [3,4]. Drive loci preferentially bias their own transmission such that a driver^+^/driver^−^ heterozygote will pass the driver^+^ allele to more than half of its viable progeny. Drivers are diverse and can act during meiosis, gametogenesis, or post-fertilization [3,5]. Both transposable elements and drivers are found throughout eukaryotes, including humans [4,5].

How do selfish genes shape sexual reproduction if most genetic loci follow Mendel’s law? As an analogy, consider human societies. Most people are law abiding, but some gain considerable personal advantages by breaking the law. This lawbreaking can exact a high cost from society. Societies, therefore, devote an oversized fraction of our social institutions and resources to deterring, apprehending, and punishing lawbreakers. This policing of lawbreakers is inextricably woven into our lives and shapes the things we do each day, generally not for the better. In fact, the policing of lawbreakers requires us all to accept unpleasant tradeoffs. Consider, for example, air travel. We surrender some of our time, privacy, convenience, and money for safety procedures designed to thwart lawbreakers. An alien being visiting earth to study our societies could draw incorrect inferences about much of our world if they failed to appreciate that lawbreakers exist. Such an alien may conclude, for instance, that humans preparing for air travel enjoy waiting in long lines for the privilege of having strangers scan their bodies and examine their personal belongings.

Similar to the situation in human societies, most genes follow Mendel’s law of segregation and are passed on to half the progeny of a heterozygote. Selfish genes, like transposable elements and drivers, can gain considerable evolutionary advantages by breaking Mendel’s law. In fact, breaking the law allows selfish genes, which often have no useful purpose, to persist and spread within genomes and within populations [3]. This selfish behavior is generally costly to the overall health and fitness of an organism. For example, transposable elements can disrupt genes, and drivers often act by destroying gametes or embryos that do not inherit them [3–5]. Therefore, it follows that genomes are selected to devote considerable resources toward thwarting genetic lawbreakers. Like the policing of human societies, this policing of genetic lawbreakers likely involves costly tradeoffs where critical pathways can be compromised [6]. Nonetheless, biologists too often neglect to consider selfish genes or the policing systems that exist to keep them in check. This can lead to misunderstandings akin to the misguided alien mentioned above. For example, the *pha-1* gene was initially misclassified as essential for development in the nematode *Caenorhabditis elegans* [7]. In reality, *pha-1* encodes a zygotically expressed antidote to a maternally deposited toxin encoded by a linked gene, *sup-35*. Together, *pha-1/sup-35* comprise a selfish drive locus that can break Mendel’s law by killing offspring that do not inherit it [8].

What mysteries of biology might be revealed via study of selfish genes and their defense systems? There are likely many, but here I will highlight 1 mystery, the paradoxical evolution of centromeres and centromere proteins [9]. The overall process of chromosome segregation is largely conserved throughout eukaryotes, but centromeres and their proteins can be rapidly evolving. In fact, natural selection often favors novel mutations in many key centromere proteins. Why would novelty be favored in genes carrying out a conserved process? One hypothesis gaining increased experimental support in a wide range of species is that rapid centromere evolution can reflect a genetic arms race between selfish driving centromeres and their defense systems, including centromere proteins [9,10]. In such arms races, novel mutations in selfish loci can give them an advantage. This, in turn, leads to selection of novelty in the defense systems to reestablish suppression of selfish genes. These adaptations to suppress selfish genes could come with tradeoffs that compromise health and fertility.

Going forward, modern biologists can follow the example set forth by early *Drosophila* geneticists and carefully consider exceptions to Mendel’s law. Such a perspective will undoubtedly yield novel insights and will likely be essential for understanding the mechanisms of heredity.
